# Analysis of the Effect of Transition-Metal Oxide Content on Reducing the Flammability of Polypropylene

**DOI:** 10.3390/polym17202734

**Published:** 2025-10-12

**Authors:** Jacek Iwko, Beata Anwajler, Roman Wróblewski, Oliwia Trzaska, Przemysław Postawa, Tomasz Jaruga

**Affiliations:** 1 Faculty of Mechanical Engineering, Wroclaw University of Science and Technology, 27 Wybrzeze Wyspianskiego Street, 50-370 Wroclaw, Poland; jacek.iwko@pwr.edu.pl (J.I.); r.m.wroblewski@pwr.edu.pl (R.W.); oliwia.trzaska@pwr.edu.pl (O.T.); 2 Department of Energy Conversion Engineering, Faculty of Mechanical and Power Engineering, Wroclaw University of Science and Technology, 27 Wybrzeze Wyspianskiego Street, 50-370 Wroclaw, Poland; 3 Faculty of Mechanical Engineering and Computer Science, Czestochowa University of Technology, Aleja Armii Krajowej 21, 42-224 Czestochowa, Poland; przemyslaw.postawa@pcz.pl (P.P.); tomasz.jaruga@pcz.pl (T.J.)

**Keywords:** polypropylene, metal oxides, flame retardancy, intumescent coatings, intumescent flame retardant (IFR), zinc oxide (ZnO), manganese oxide (MnO)

## Abstract

Polypropylene (PP) exhibits high flammability (LOI ≈ 17.5%), which limits its industrial applications. Previous studies have primarily focused on the flame-retardant mechanisms of intumescent flame-retardant (IFR) systems, while less attention has been given to the role of inorganic synergistic additives in balancing flame retardancy with mechanical performance—an aspect crucial for commercial applications This study investigated the effect of small additions of zinc oxide (ZnO) and manganese oxide (MnO) on the flame-retardant, mechanical, and thermal properties of PP/IFR (APP + PER) composites. The oxide content was varied between 0 and 2 wt.%. LOI and UL-94 tests showed that as little as 0.25 wt.% increased LOI to 30% and enabled all materials to achieve a UL-94 V-0 classification. The highest performance was observed for ZnO (LOI = 43.7% at 1.5 wt.%), while MnO induced a linear increase up to 38.6% at 2 wt.%. SEM analysis confirmed the formation of a compact, foamed char layer. Mechanical testing revealed improved stiffness (~15%) and flexural strength (~20%), with unchanged tensile strength but reduced impact strength (−50% for ZnO, −30% for MnO). The HDT increased from 55 °C to 65 °C. These findings demonstrate that small amounts of ZnO and MnO act as effective and economically viable IFR synergists in PP composites.

## 1. Introduction

Polypropylene (PP) is a polyolefin and one of the most widely used thermoplastic polymers worldwide. Its annual production exceeds 80 million tons, and its share of the polymer market continues to grow. PP’s popularity stems from its favorable attributes: low density, high chemical resistance, ease of processing, good mechanical performance, and cost effectiveness [1]. Polypropylene is widely used in the automotive industry for interior components, battery housings, and structural parts. It is also used in electrical and electronic applications for equipment housings and wire insulation. Additionally, polypropylene is used in construction for pipes, profiles, and insulating boards, as well as in the packaging and medical/healthcare sectors.

Despite its many advantages, polypropylene’s pronounced flammability is a major limitation to its broader use in applications requiring high fire resistance. Polypropylene’s limiting oxygen index (LOI) is only about 17.5%, indicating that the material readily burns in ambient air, which contains 21% oxygen [1]. Furthermore, polypropylene produces toxic combustion products that can be released in large quantities during a fire, posing risks to humans and the environment. These limitations make developing effective flame-retardant strategies for polypropylene an active area of intensive academic and industrial research.

Many strategies have been developed to reduce the flammability of polypropylene and other polymers. One such strategy is the use of halogenated additives, the most effective flame-retardant approach for many years [2]. However, brominated or chlorinated flame retardants are being phased out due to the toxic compounds they emit during combustion. Another approach is endothermic dehydration, which involves incorporating metal hydroxides (Al(OH)_3_ and Mg(OH)_2_) in suitable forms. These hydroxides act as physical barriers, releasing water and cooling the material’s surface during thermal decomposition [3]. However, this solution requires very high loadings (40–60 wt%) to be effective, which markedly deteriorates the polymer’s mechanical properties. A more favorable strategy is to use phosphorus–nitrogen compounds, which act in both the condensed and gas phases. These compounds reduce the emission of flammable pyrolysis products and inhibit combustion chain reactions [4]. Intumescent systems (IFRs—intumescent flame retardants) have also been employed and have become particularly attractive in recent years. Upon heating, they foam and form a char layer on the surface of the material that shields the polymer substrate from flames and heat.

Each of the above approaches has inherent limitations. Consequently, there is a growing interest in using inorganic additives, such as transition-metal oxides, in conjunction with IFR systems and phosphorus-based flame retardants [5,6,7,8]. These additives can improve the effectiveness and stability of these systems.

### The Role of Transition-Metal Oxides in the Process of Flame Retardance

Transition-metal oxides, such as iron(III) oxide (Fe_2_O_3_), zinc oxide (ZnO), and titanium(IV) oxide (TiO_2_), are of particular interest in technologies aimed at reducing the flammability of polypropylene [1]. The modes of action of these oxides can be characterized on several levels. One level (I) is char-forming catalysis. These oxides accelerate the polymer’s dehydration and condensation reactions, forming a stable char layer on its surface (Fe_2_O_3_, ZnO, and MnO_2_) [1]. (II) Thermal stabilization: These raise the onset temperature of polypropylene degradation, meaning pyrolysis begins at higher temperatures (TiO_2_ and Co_3_O_4_) [3]. (III) Formation of inorganic barriers: In the presence of metal oxides, protective ceramic layers (e.g., metal phosphates) form, which exhibit low thermal conductivity and high oxidation resistance (ZnO and Fe_2_O_3_) [3]. (IV) Reduction in flammable gas release: By modifying the degradation pathway, these oxides decrease the quantity of volatile hydrocarbons in the gas phase, which attenuates combustion and certain physical effects (TiO_2_). The oxide reflects and scatters thermal radiation, limiting sample heating [9].

In addition, the interaction between the metal oxides and the PP matrix is most likely of a physicochemical nature. At the nanoparticle level, there is a reduction in the mobility of polymer chains, which affects the mechanical properties of the composite. On the one hand, at the nanoparticle level, there is a reduction in the mobility of polymer chains, which affects the mechanical properties of the composites. Work [10] demonstrated that the presence of MnO_2_ nanoparticles in the polypropylene matrix decreases the crystallinity of the polymer composite (even at nanofiller contents as low as 0.5 and 1.0%). Relatively small contents of metal oxide nanofillers also favorably enhance the thermal stability of the polypropylene matrix and may contribute to preventing the formation of burning drips [11]. Study [12] showed that the addition of ZnO at 2.0% within the PP matrix resulted in a reduction in the Young’s modulus (relative to neat PP), but after 24 weeks of exposure to sunlight, this modulus was at a higher level compared to the un-filled samples. Thus, ZnO exhibited photocatalytic properties and contributed to the reduced degradation of the polymer matrix material under solar radiation. Hence, the presence of metal oxides in the PP matrix can act synergistically by influencing mechanical and thermal properties, and may also exert a catalytic effect.

Studies also indicate that the effectiveness of transition-metal oxides depends on their type, particle size (micro- vs. nanoparticles), degree of dispersion within the polymer matrix, and interactions with other additives [5]. For example, TiO_2_ nanoparticles exhibit not only a catalytic effect but also a physical one—they limit heat penetration into the material via thermal-radiation scattering. In summary, Table 1 presents selected literature data for PP/IFR (APP/PER) systems containing ZnO, TiO_2_, or MnO_2_ (various forms), including LOI, UL 94 V, pHRR, and THR values. Table 2 lists representative loading ranges of transition-metal oxides in PP-based composites.

In summary, the results of studies conducted to date suggest that Fe_2_O_3_ and ZnO are among the most effective additives for reducing the peak heat release rate (pHRR) and increasing the limit of ignition (LOI) [2]. TiO_2_ acts in both chemical and physical ways, making it suitable for hybrid systems [9]. Meanwhile, MnO_2_ and CuO reduce smoke and toxic gas emissions, and Co_3_O_4_ and Cr_2_O_3_ strengthen the charred barrier [1]. However, the effectiveness of these additives depends on the quality of dispersion. The best results are achieved in multi-component systems containing IFR and phosphorus additives, with LOI values of over 28% and UL-94 V-0 classification [3,5]. For instance, Deng et al. [16] demonstrated that combining microencapsulated IFR with silanized ZnO can increase the LOI to 39.8%, achieve V-0 classification, and reduce pHRR, TSP, and gas emissions while enhancing water resistance. Li et al. [18] used a Mg(OH)_2_ hybrid containing ZnO nanoparticles, which reduced pHRR and gas emissions while improving mechanical properties. Feng et al. [22] demonstrated that adding 1% MnO_2_ significantly increases the LOI, reducing the pHRR, THR, and SPR whilst stabilizing the char layer. Dong et al. [14] demonstrated the synergistic effect of APP with HP-Mn, achieving an LOI of 30.7% and UL-94 V-0 classification, whilst reducing pHRR by 74%, compared to pure PP. SEM and Raman analysis confirmed the formation of a compact, highly ordered char layer.

Such additives can enhance fire safety and restrict the spread of fire in practical applications, such as in automotive components (Fe_2_O_3_ and ZnO), electronics (TiO_2_), building materials (MnO_2_ and CuO), and in the transportation and aviation industries (Co_3_O_4_ and ZnO).

In summary, the high flammability of polypropylene and the limited effectiveness of traditional flame-retardant methods mean solutions based on IFR systems with transition-metal oxide additives are becoming increasingly important. While previous studies have primarily focused on the mechanism of action of these systems and their impact on thermal properties, their consequences for mechanical properties—which determine the material’s industrial applicability—have received less attention.

This study, therefore, examines the impact of minor additions of ZnO and MnO on the flammability and mechanical properties of PP/IFR composites. The economic aspect has also been considered; the additives used are inexpensive and widely available, potentially making the proposed solutions competitive in industrial settings. This research includes an analysis of changes in mechanical and flame-retardant parameters as a function of oxide content, as well as a detailed characterization of the morphology of charred layers using scanning electron microscopy (SEM). This has enabled the identification of the optimal conditions in which a balance is maintained between improved fire resistance and acceptable mechanical properties.

## 2. Materials and Methods

### 2.1. Materials

**Figure 1:**
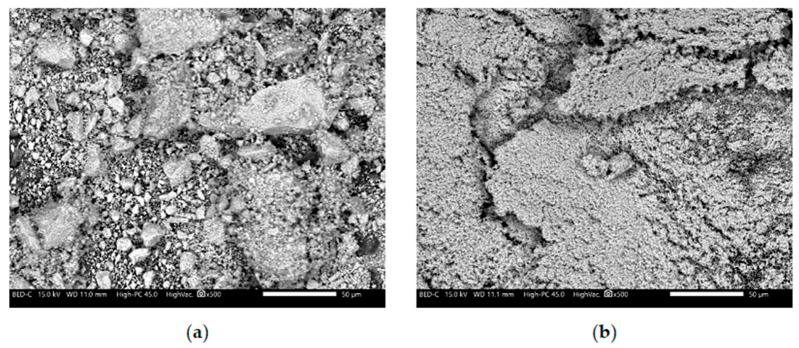
SEM images of (**a**) manganese oxide and (**b**) zinc oxide in magnification ×500.

The development of novel, non-flammable polypropylene-based materials that form an intumescent protective layer during combustion relied on the following components: The matrix material was polypropylene (PP) HP Moplen 456J (Basell Orlen Polyolefins, Płock, Poland) with a medium melt flow rate (MFR) of 3. The gas source was ammonium polyphosphate (APP) APP1201 (WTH GmbH, Stade, Germany) and the carbon source was pentaerythritol (PER) A10520 (ThermoFisher GmbH, Frankfurt, Germany). The catalysts for forming the intumescent layer were metal oxides (main group and transition-metal oxides), specifically zinc oxide (ZnO) and manganese(II) oxide (MnO). Both were analytical grade (Chempur). SEM images of the metal oxides used in this study are presented in Figure 1. The metal oxides used have a nanometric structure. In the case of MnO, a clear tendency for particle agglomeration is observed (Figure 1a), while ZnO is characterized by a much finer dispersion without visible agglomerates (Figure 1b). The metal oxides used were not subjected to any surface treatment prior to their incorpo.

Based on the results of previous studies [7,8], the baseline formulation composition was assumed to be PP: 73–75%; APP: 15%; PER: 10%; and MO: 0–2%.

This study focused on preparing materials containing varying amounts of specific metal oxides. Composites containing 0.25%, 0.50%, 0.75%, 1.0%, 1.5%, or 2.0% by weight of each metal oxide were produced. Systems incorporating zinc oxide (ZnO) and manganese(II) oxide (MnO_2_) were selected for investigation. For powder composites, not only is the filler loading in the polymer matrix significant, but also the size of the added particles. In the case of nanofillers (such as the ZnO and MnO metal oxide powders introduced in this study), their high surface-to-volume ratio enables them to be incorporated into the polymer matrix in a relatively small amount. This low loading is sufficient to reduce the mobility of polymer chains and improve the mechanical properties of the polymer composite [10,25]. In the present work, filler concentrations ranging from 0% to 2% were proposed, which is consistent with generally accepted standards. These materials were labeled Z0–Z6 and M0–M6, and their compositions are summarized in Table 3. The primary factor driving the selection of the composite components was their relatively low cost. This is particularly relevant given that in industrial settings the choice of a material is often determined by its cost and availability, frequently superseding purely functional properties. The objective of this study is therefore to propose material solutions that are both cost effective and economically feasible for industrial-scale applications.

The mixtures were prepared by compounding PP granules with APP and PER, followed by the addition of the respective metal oxide. Then, the respective metal oxide was added. After thorough mixing, the blends were dried in a hot-air dryer at 105 °C for 48 h until the moisture content was below 0.05%. The prepared and dried mixtures were then processed using a co-rotating twin-screw extruder (manufacturer: Zamak Mercator) with a screw diameter of D = 24 mm, an L/D ratio of 40, and ten heating zones. Extrusion was carried out under the following conditions: screw speed: 60 rpm; barrel temperature profile across successive heating zones: 160 °C, 180 °C, 185 °C, 190 °C, 190 °C, 195 °C, 200 °C, 200 °C, and 190 °C. The die temperature was 190 °C.

Test specimens were prepared using a Battenfeld Plus 350/75 screw injection molding machine with the following specifications: screw diameter (D) = 25 mm; screw length-to-diameter ratio (L/D) = 17; and number of flights in zones = 14/4/4. The key parameters of the injection molding process are summarized in Table 4.

As a result of the injection molding process, the following specimens were obtained:

(a) dumbbell specimens, 150 × 10 × 4 mm, for mechanical property testing;

(b) bars, 130 × 13 × 4 mm, for flammability testing (UL-94V);

(c) bars, 80 × 10 × 4 mm, for flammability (LOI), impact strength, and HDT testing.

### 2.2. Methods

The produced composites were subjected to tests determining the limiting oxygen index (LOI), UL-94 flammability classification, tensile strength, flexural strength, unnotched Charpy impact strength, heat deflection temperature (HDT), and SEM analysis.

#### 2.2.1. Limiting Oxygen Index (LOI)

LOI was measured using an oxygen index tester, type OI (Advanced Instruments, China), in accordance with ISO 4589-2:2017 [26]. The test specimens had dimensions of 80 × 10 × 4 mm.

#### 2.2.2. UL-94V Test

The vertical UL-94V test was carried out using a Testex TF328 UL94 H&V Flammability Tester, in accordance with the UL-94 test standard [27], Table 5. Specimens with dimensions of 130 × 13 × 4 mm were used. Ten specimens of each material were tested.

#### 2.2.3. Heat Deflection Temperature (HDT)

The heat deflection temperature of the tested materials was determined using a VICAT-HDT AUTOMATIC apparatus (Monagraph Studio, Kęty, Poland). Measurements were performed according to the PN-EN ISO 75-2:2013-06 [28] standard, under a load of 0.45 MPa and with a heating rate of 120 °C/h.

#### 2.2.4. Mechanical Properties

The flexural modulus and flexural strength of the composites were measured using a Tinius Olsen H25KT universal testing machine equipped with a 10 kN load cell. Tests were carried out at room temperature at a crosshead speed of 50 mm·min^−1^, using at least eight standard dumbbell-shaped specimens for each material type.

#### 2.2.5. Impact Strength

The Charpy impact strength of the fabricated specimens was measured using a Resil 5.5 impact tester (Ceast). A hammer with an energy of 2 J was applied, and ten specimens from each series were tested. Only unnotched impact strength was measured, in accordance with ISO 179-1/1eU [29]. During the test, the specimens were positioned edgewise, with a support span of 62 mm.

#### 2.2.6. Scanning Electron Microscopy (SEM)

The residues of burned specimens were examined for differences in the appearance and external structure of the formed char using a Hitachi TM3000 scanning electron microscope equipped with a SwiftED3000 EDS system, enabling surface observation, analysis, and characterization of morphology and elemental composition. The samples were not sputter-coated with a conductive layer, as the char exhibited sufficiently good electrical conductivity for the purpose of this analysis.

Additionally, SEM images of metal oxide samples (ZnO and MnO—Figure 1) were taken at 500× magnification using a JEOL JSM-IT210 scanning electron microscope (SEM) with backscattered electrons.

## 3. Results and Discussion

**Figure 2:**
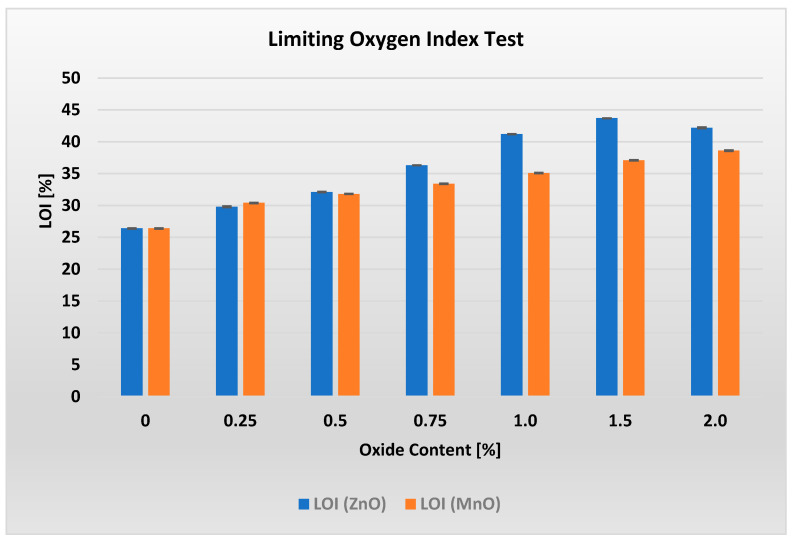
Results of limiting oxygen index (LOI) measurements for materials containing different amounts of zinc oxide (ZnO) and manganese oxide (MnO).

This section presents the results of all conducted tests together with a brief discussion. The limiting oxygen index (LOI) values for materials containing different amounts of ZnO and MnO are shown in Figure 2.

Analysis of Figure 2 reveals the existence of an optimal ZnO content of 1.5%, at which the LOI reaches its maximum value of 43.7. This represents the highest oxygen index measured throughout the entire study.

Analysis of results shown on Figure 2 shows that adding increasing amounts of MnO results in an almost linear increase in the LOI value, from 30.4% for 0.25% MnO to 38.6% for 2% MnO. In this case, unlike the ZnO-containing systems, no maximum LOI was observed. This suggests that there may be a higher MnO content in the PP/APP/PER/MnO system at which the LOI reaches its maximum.

All tested materials, even at the minimum metal oxide content of 0.25%, proved to be completely non-flammable (UL-94 V-0), similar to the reference material (Z0/M0) with zero metal oxide content.

**Figure 3:**
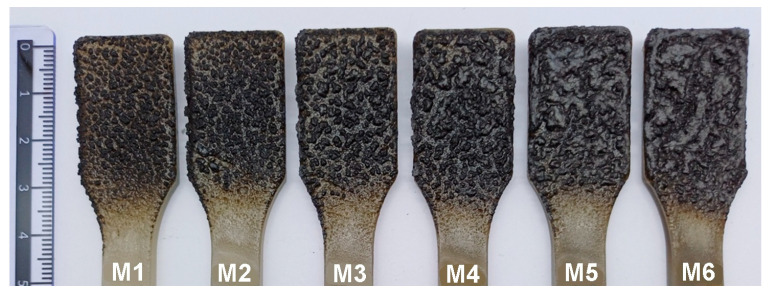
Char residues of materials containing different amounts of MnO.

The material characterization was extended to include macroscopic photographs of the coatings after sample combustion, as considerable differences in the appearance of the char residues were observed that were not evident in the SEM images. The samples were burned using a Topex standard burner with a fixed flame length of 80 mm, positioned at a distance of 50 mm from the nozzle. The combustion process lasted 15 s from the moment the flame was applied to the specimen. The upper part of the dumbbell-shaped sample (the widened grip section used for mechanical testing) was subjected to combustion. Macroscopic photographs of the char residues of samples M1–M6 (containing manganese oxide, MnO, in amounts of 0.25%, 0.5%, 0.75%, 1%, 1.5%, and 2%, respectively) are presented in Figure 3.

Figure 3 shows that the char samples M1 and M2 (containing 0.25% and 0.50% MnO, respectively) consist of small, unconnected carbon particles. In contrast, the chars of samples M3–M6 reveal small carbon islands merging into larger, increasingly irregular clusters. These differences are reflected in the SEM micrographs of these materials’ chars. Images of the char of samples Z1–Z6 are not presented because the differences between them are similar to those of M1–M6, as shown in Figure 2, and do not provide additional insight for the analysis.

The flame-retardancy mechanism of PP through the addition of MnO may be based on enhancing the strength of the char residue formed during combustion, as a result of the catalysis of PP oxidative dehydrogenation by the metallic moieties, which leads to partial matrix cross-linking. Another suggested mechanism involves the formation of a ceramic barrier during combustion, which limits heat and mass transfer [11]. Similarly, ZnO is attributed with a catalytic mechanism that promotes the dehydrogenation and oxidation of PP, thereby increasing the density and cross-linking of the resulting char [30]. Therefore, both applied oxides exhibit a catalytic effect in the PP combustion process and favor the formation of a compact char structure, which acts as a physical barrier during the combustion process.

**Figure 4:**
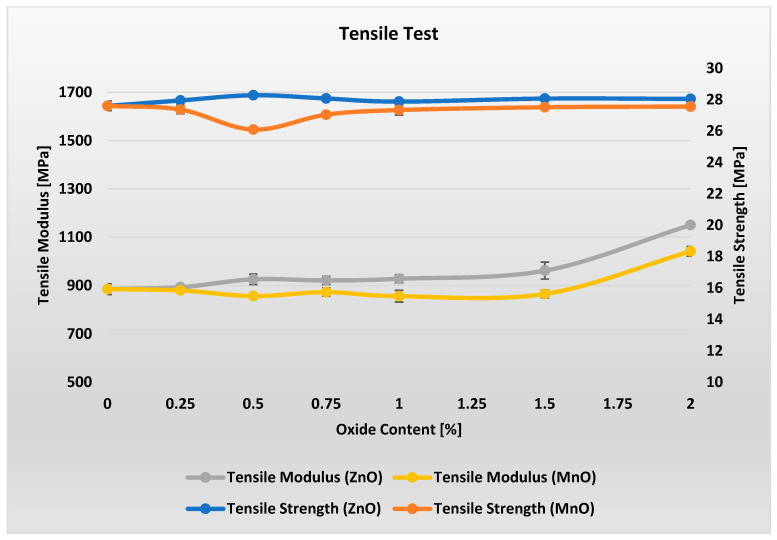
Results of tensile test measurements for varying ZnO and MnO content.

Figure 4 present the results of tensile property tests for materials with varying metal oxide content.

Analysis of Figure 4 shows that the tensile strength of materials with varying metal oxide content in the range of 0–2% remains essentially unchanged. For MnO-containing materials, a slight minimum in Rm can be observed. However, when considering all results within each measurement series along with the error bars, it can be concluded that ZnO and MnO contents in the range of 0–2% have virtually no effect on the tensile strength of these materials.

Analysis of the stiffness modulus indicates that adding metal oxides in the range of 0.25–1.5% has virtually no effect on the stiffness of the materials. Only a noticeable change is observed at a loading of 2% metal oxide in the PP/APP/PER system, where stiffness increases by approximately 15%.

**Figure 5:**
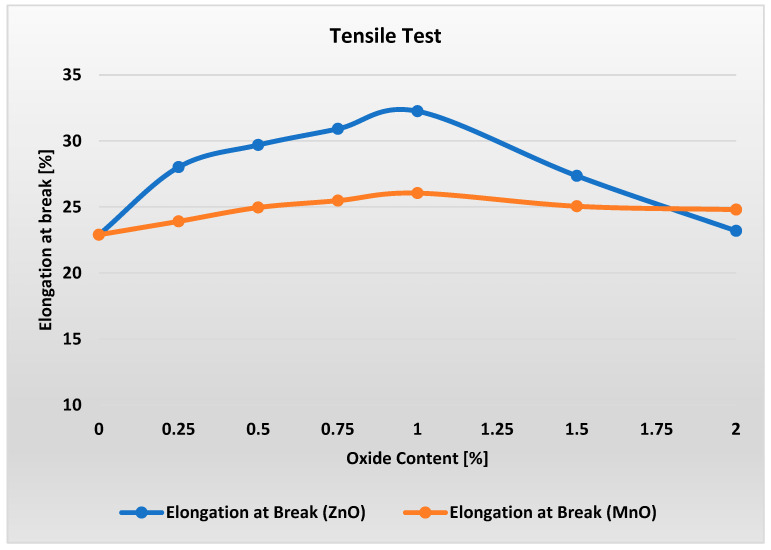
Results of elongation at break measurements for varying ZnO and MnO content.

**Figure 6:**
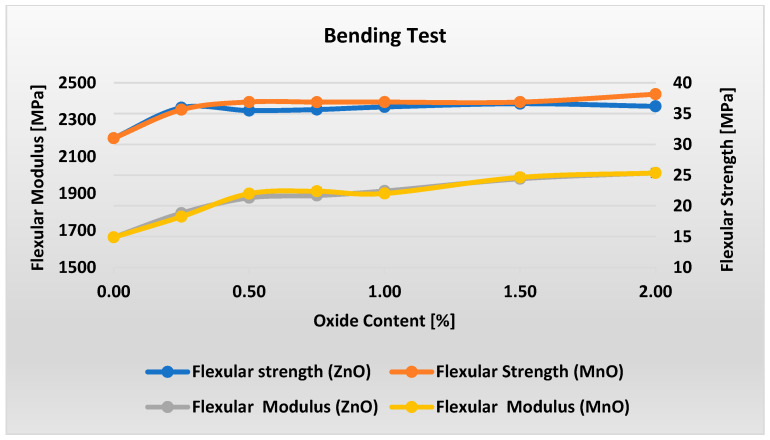
Results of flexural strength measurements for varying ZnO and MnO content.

Figure 5 shows a distinct peak in the elongation at break of the material containing ZnO at oxide content levels of about 1%. For this content, the material exhibits about 4% higher elongation at break compared to raw material but finally ends up with a result close to that of raw material. Small but distinct peaks can be observed also in the elongation at break of the material containing MnO at oxide content levels of 0.75–1%. For 2% content, elongation at break is higher than for ZnO and raw material. Figure 6 presents the flexural strength and modulus test results for materials with varying metal oxide content in the PP/IFR/MO system.

Analysis of Figure 6 shows that adding even a small amount of metal oxide increases the material’s flexural strength. For ZnO-containing materials, increasing the oxide content beyond 0.25% has virtually no effect on flexural strength, which remains constant at 36 MPa within the measurement uncertainty. For MnO-containing materials, an initial, pronounced increase in flexural strength is observed up to 0.5% oxide. Subsequently, samples M2 and M5 exhibit a constant value of 36.9 MPa. Only at 2% MnO is a slight, approximately 4% increase observed. Regardless of the type of metal oxide used, increasing its content in the material from 0 to 2% results in a slight, nearly linear increase of approximately 20% in flexural modulus.

**Figure 7:**
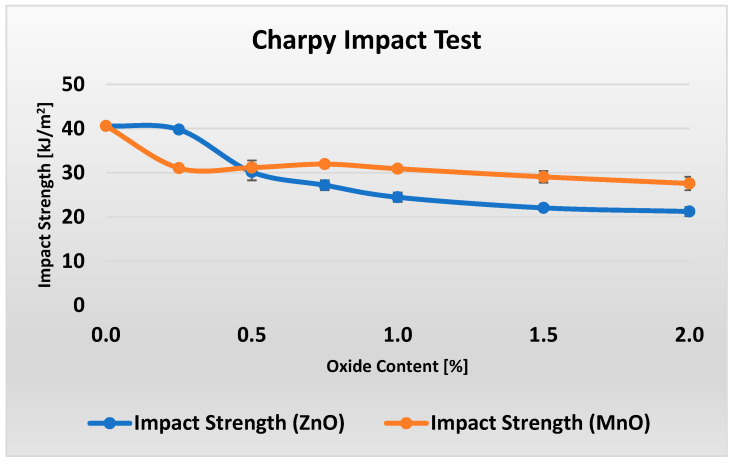
Results of unnotched Charpy impact strength measurements for varying ZnO and MnO content.

Figure 7 shows the unnotched Charpy impact strength results for composites with different metal oxide content.

Figure 7 shows that increasing the ZnO content in the material considerably reduces its impact strength by about 50% within the 0–2% range. The decrease in impact strength for MnO is less pronounced, at around 32%. Examining the data more closely reveals that, when material M0 (0% MnO) is excluded, the impact strength of samples M1–M6 (0.25–2% MnO) remains essentially constant within the measurement uncertainty. It is worth noting that the addition of 0.25% ZnO (in contrast to the same amount of MnO) had virtually no effect on the impact strength of the material, while improving its flame-retardant performance by 4% in the LOI test and enhancing flexural strength by 20%.

**Figure 8:**
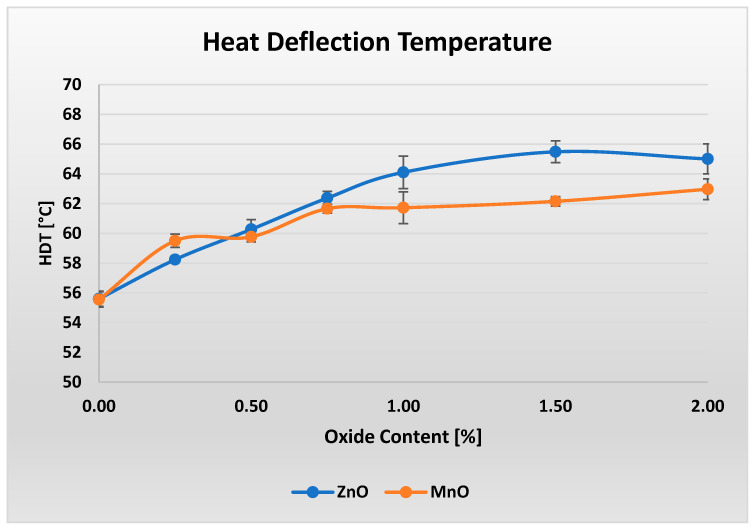
Results of heat deflection temperature (HDT) measurements for varying ZnO and MnO content.

Figure 8 shows the heat deflection temperature (HDT) results for materials containing varying amounts of oxide, ranging from 0% to 2%.

Although the changes in HDT are relatively small, they are clearly visible in both cases. Materials containing ZnO exhibit a slight, nearly linear increase in HDT up to 1.5% ZnO content, followed by a minor decrease at 2% ZnO. Overall, the variation in HDT amounts to approximately 10 °C across the entire investigated range. In contrast, a modest increase in HDT of about 7.5 °C is observed for the materials containing MnO between the lowest and highest MnO loadings.

**Figure 9:**
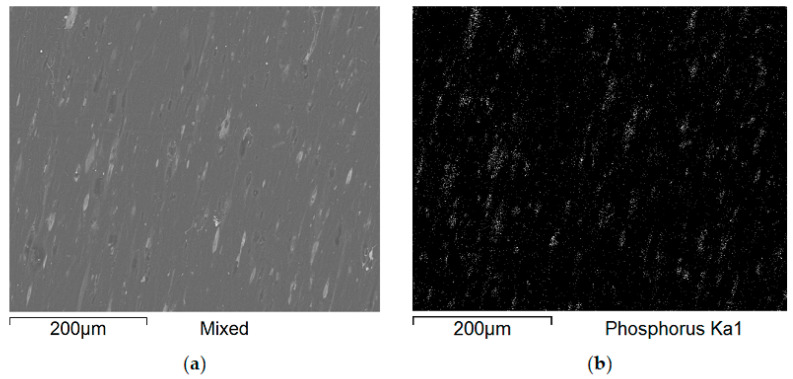
SEM pictures (**a**) and EDS maps (**b**) of the distribution of phosphorus in not-burned specimen surfaces without manganese oxide.

**Figure 10:**
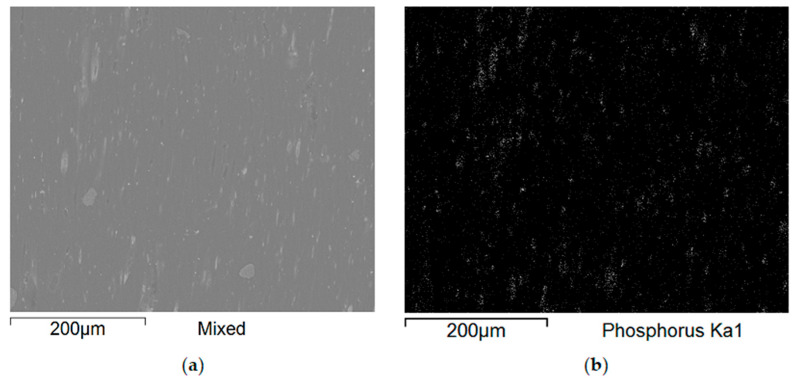
SEM pictures (**a**) and EDS maps (**b**) of the distribution of phosphorus in not-burned specimen surfaces containing 2.00% manganese oxide.

Additional SEM images of the samples were taken prior to the flammability tests (Figure 9 and Figure 10). Unfortunately, the photos are very similar and do not provide much relevant information. This paper includes illustrative SEM and EDS (phosphorus) images of samples M0 and M6. These images demonstrate that the bright spots visible in the SEM images are phosphorus compounds (APP). The incorporation of MnO particles into the PP matrix reduces the size of voids, which appear as darker, elongated objects in the SEM images. This also enhances the comminution of the phosphorus compound (present as the APP additive).

Figure 9 and Figure 10 present a comparison of EDS analysis maps of the surface of samples without MnO addition and those containing 2.00% MnO. The visible bright spots, for which EDS analysis confirmed the presence of phosphorus and oxygen, show significant refinement in the 2.00% MnO sample compared to the MnO-free sample.

**Figure 11:**
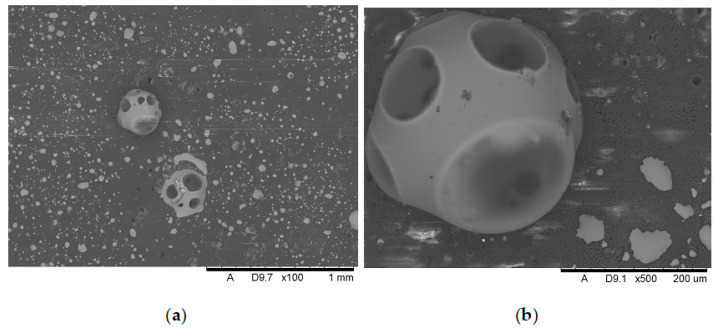
Scanning electron microscope (SEM) images of burned specimen surfaces without manganese oxide: (**a**) magnification ×100, (**b**) magnification ×500.

**Figure 12:**
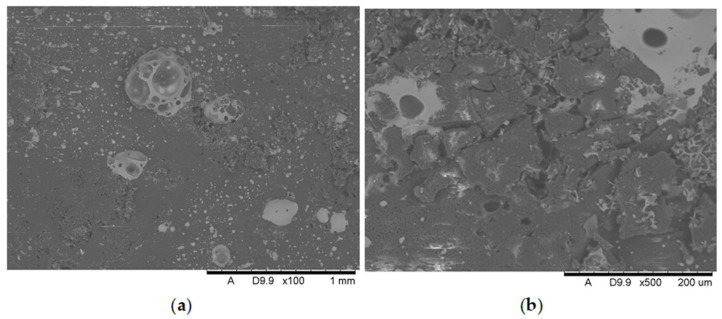
Scanning electron microscope (SEM) images of burned specimen surfaces containing 0.25% manganese oxide: (**a**) magnification ×100, (**b**) magnification ×500.

**Figure 13:**
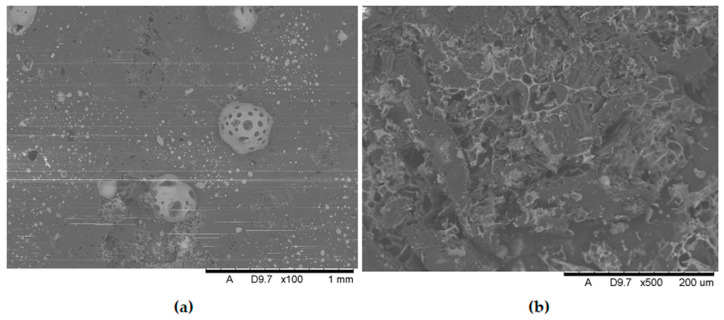
Scanning electron microscope (SEM) images of burned specimen surfaces containing 0.5% manganese oxide: (**a**) magnification ×100, (**b**) magnification ×500.

**Figure 14:**
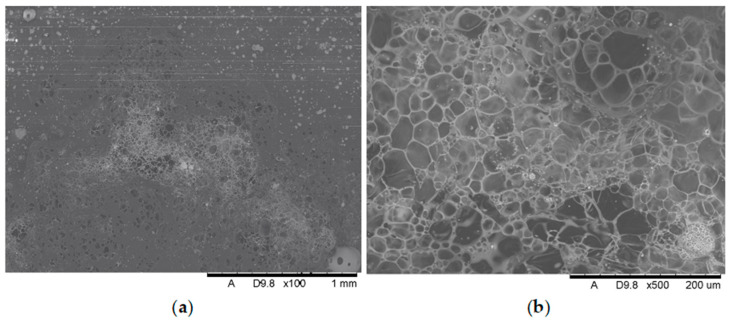
Scanning electron microscope (SEM) images of burned specimen surfaces containing 0.75% manganese oxide: (**a**) magnification ×100, (**b**) magnification ×500.

**Figure 15:**
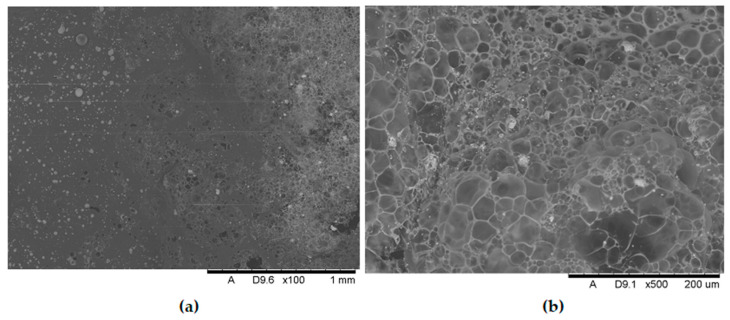
Scanning electron microscope (SEM) images of burned specimen surfaces containing 1% manganese oxide: (**a**) magnification ×100, (**b**) magnification ×500.

**Figure 16:**
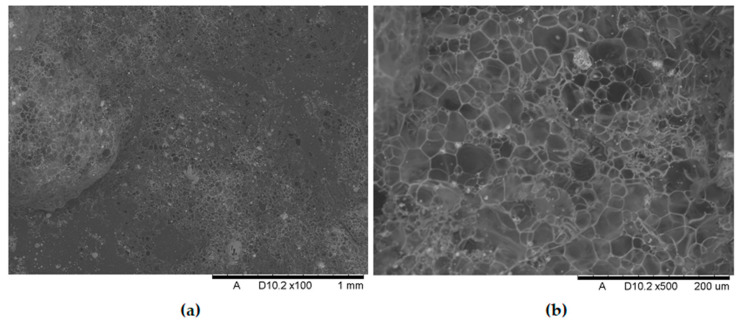
Scanning electron microscope (SEM) images of burned specimen surfaces containing 1.5% manganese oxide: (**a**) magnification ×100, (**b**) magnification ×500.

**Figure 17:**
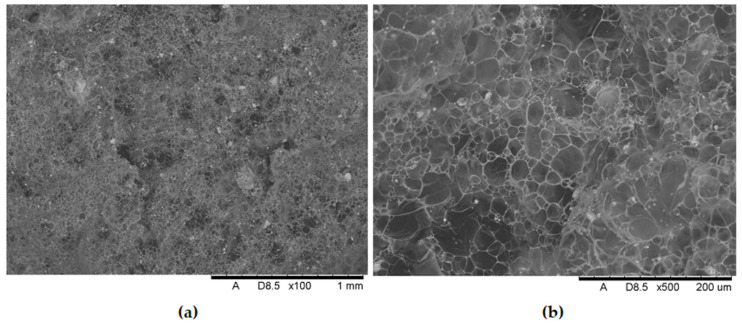
Scanning electron microscope (SEM) images of burned specimen surfaces containing 2% manganese oxide: (**a**) magnification ×100, (**b**) magnification ×500.

Figure 11, Figure 12, Figure 13, Figure 14, Figure 15, Figure 16 and Figure 17 show scanning electron microscope (SEM) images of burned specimens containing manganese oxide. Images of the charred surfaces of materials containing MnO are shown, as the images of systems containing ZnO were very similar.

Analysis of the images in Figure 11, Figure 12, Figure 13, Figure 14, Figure 15, Figure 16 and Figure 17 (at 100× magnification) clearly shows an increase in foamed char content as the MnO concentration in the material increases. The foamed char phase is absent up to an LOI value of 30% and begins to appear at an LOI value of approximately 32%.

Figure 11, Figure 12, Figure 13, Figure 14, Figure 15, Figure 16 and Figure 17 display the characteristic structures of phosphorus oxides, forming porous spheres. These spheres are observed in both the metal oxide-free samples and the nanocomposites. However, the number of these spheres decreases with an increasing percentage of metal oxides. For composites containing 0.75% metal oxide, these structures are practically no longer observed; instead, a characteristic foamed char appears (which is absent in materials with lower metal oxide content). It is noteworthy that the aforementioned foamed char is present in samples exhibiting an LOI index above 30%. Thus, it can be inferred that the presence of the foamed char layer effectively isolates the sample surface from oxygen access, which favorably contributes to their flame retardancy.

## 4. Conclusions

This study evaluated the flammability and mechanical performance of polypropylene (PP)-based materials incorporating two key components of intumescent coatings: ammonium polyphosphate (APP) and pentaerythritol (PER). We examined novel PP-APP-PER systems containing two transition-metal oxides: zinc oxide (ZnO) and manganese oxide (MnO). We varied the oxide content from 0 to 2%. Then, we conducted flammability tests alongside mechanical and thermal analyses to determine the materials’ flame-retardant, mechanical, and thermal properties. We also conducted structural characterization using scanning electron microscopy (SEM).

An increase in the content of ZnO and MnO in the PP/IFR/MO system confirms the hypothesis that this leads to a gradual increase in the LOI index. The LOI increases approximately linearly with the metal oxide content. However, the materials remain non-flammable (UL-94 V-0) regardless of the oxide loading across the entire investigated range. Notably, even a minimal MO content (0.25%) significantly reduces flammability (by 4% LOI) while improving flexural strength, flexural modulus, and HDT.

Impact tests were performed on the samples prior to burning. The impact strength values and curve profiles for both types of sample were similar. In the case of MnO, the filler has a wide particle size distribution, so changing the amount of this filler does not significantly affect the mechanical properties, as can be seen in the tensile modulus and elongation at break graphs. In the case of ZnO, the particle size distribution is more uniform, meaning that changing the amount of this filler causes greater changes in properties.

The hypothesis that higher ZnO and MnO contents in the PP/IFR system act as catalysts for forming a denser, more oxidation-resistant char layer that more effectively insulates the interior of the material from heat and oxygen sources was also confirmed, as evidenced by SEM images. Additionally, increasing MO content in the PP/IFR/MO system reduces flammability up to a certain point. However, beyond this threshold, an increase in flammability is observed, as shown in Figure 2 and reported by other researchers [7,31,32]. One reason for this phenomenon is the reinforcement of charcoal by metal oxide (MO) particles, particularly those with a high aspect ratio. While increased stiffness can improve the mechanical properties of char, such a stiff charred material can disrupt the continuity of its structure and lead to cracks in the protective shell when it becomes too stiff. This can occur when the metal oxide content exceeds the optimum amount, which varies for different MOs [6,33].

## Figures and Tables

**Table 1:** PP systems (IFR + metal oxides).

System (PP + Additives)	Composition/Loading (wt%)	LOI (%)	UL-94	pHRR (kW/m^2^)	THR (MJ/m^2^)	Cone Conditions (kW/m^2^; Sample; Standard)	Reference
PP (neat)—base	0	18.5	NR	804.3	88.8	35; 100 × 100 × 3 mm; ISO 5660-1 [13]	[14]
PP + 30% IFR (APP/PER = 2:1)	APP 20; PER 10	25.4	V-0	355.5	126.1	35; 3 mm; ISO 5660 [13]	[15]
PP + 30% IFR + 0.5% nano-SiO_2_	APP 19; PER 9.5; SiO_2_ 0.5	26.7	V-0	295.3	124.2	35; 3 mm; ISO 5660 [13]	[15]
PP + 25% (APP/HP-Mn (1:1)	APP 12.5; HP-Mn 12.5	30.7	V-0	207.5	57.0	35; 100 × 100 × 3 mm; ISO 5660-1 [13]	[14]
PP + 24% Si-IFR + 2% Si-ZnO	Si-IFR 24; Si-ZnO 2	39.8	V-0			—	[16]
PP + 40 phr KZO@MH (ZnO-doped Mg(OH)_2_)	≈40 phr			327.0		50; 100 × 100 × 3 mm; GB/T 16172-2007 [17]	[18]
PP + 30% IFR + TiO_2_ (≈1–2%)	APP/PER 2:1; TiO_2_ ~1–2					—	[6]

pHRR—peak heat release rate, THR—total heat release.

**Table 2:** Typical loading ranges.

Oxide	Role in PP/IFR	Typical Loading Range (wt%)	Typical Cone Conditions)	Example Systems/Notes	Ref.
Fe_2_O_3_ (iron(III) oxide)	char catalysis; pore sealing; smoke suppression	0.5–3	35–50 kW/m^2^; 3 mm (ISO 5660 [13])	PP/IFR; WPC + APP; optimum ≤ 2 wt%	[19,20]
ZnO (zinc oxide)	catalysis of dehydration/carbonization; IFR synergist; smoke suppression	0.5–2 (as IFR synergist); up to 40 phr in mineral formulations	35 kW/m^2^; 3 mm (ISO 5660 [13])	PP/Si-IFR/Si-ZnO (2%); KZO@MH w PP (50 kW/m^2^)	[16,18]
TiO_2_ (titanium dioxide)	inorganic barrier; char stabilization/compactness	0.5–3 (synergist)	35 kW/m^2^; 3 mm	Effects depend on morphology and dispersion	[20,21]
MnO_2_ (manganese(IV) oxide)	catalysis of cross-linking/carbonization; char reinforcement	0.5–2 (nanosheets/synergist)	35 kW/m^2^; 3 mm	~1 wt% often near LOI/pHRR optimum	[14,22]
CuO (copper(II) oxide)	oxidative catalysis; char sealing; smoke reduction	0.5–2	35 kW/m^2^; 3 mm	More frequently reported in coatings/epoxy; reference point for PP	[20,23]
Co_3_O_4_ (cobalt(II,III) oxide)	oxidative catalysis; stabilization of the char layer	0.5–1.5	35 kW/m^2^; 3 mm	IFR synergist in PP; requires good dispersion	[20,24]
Cr_2_O_3_ (chromium(III) oxide)	char catalysis; barrier densification/stiffening	0.5–1	35 kW/m^2^; 3 mm	Reports of 1 wt% →HRR reduction	[20]

**Table 3:** Compositions of formulations with varying metal oxide content.

Z0	-	75.0	15	10	0.00
Z1	ZnO	74.75	15	10	0.25
Z2	ZnO	74.5	15	10	0.50
Z3	ZnO	74.25	15	10	0.75
Z4	ZnO	74.0	15	10	1.00
Z5	ZnO	73.5	15	10	1.50
Z6	ZnO	73.0	15	10	2.00
M0	-	75.0	15	10	0.00
M1	MnO	74.75	15	10	0.25
M2	MnO	74.5	15	10	0.50
M3	MnO	74.25	15	10	0.75
M4	MnO	74.0	15	10	1.00
M5	MnO	73.5	15	10	1.50
M6	MnO	73.0	15	10	2.00

**Table 4:** Injection molding parameters.

Parameter	Value
Temperature of heating zones 1, 2, 3	200, 205, 205 [°C]
Mold temperature	30 [°C]
Screw rotation speed	120 [rpm]
Injection pressure	850 [bar]
Holding pressure	350 [bar]
Holding time	12 [s]
Cooling time	18 [s]

**Table 5:** Vertical Flammability test standards and characteristics (UL-94V).

Criteria Conditions	V-0	V-1	V-2
Afterflame time for each individual specimen t_1_ and t_2_	<10 s	<30 s	<30 s
Total afterflame time for any condition set (t_1_ + t_2_ for all 5 specimen)	<50 s	<250 s	<250 s
Afterflame plus afterglow time for each individual specimen after the second flame application (t_2_ + t_3_)	<30 _S_	<60 s	<60 s
Afterflame or afterglow of any specimen up to the holding clamp	No	No	No
Cotton indicator ignited by flaming particles or drops	No	No	Yes

t_1_—the total burning time of the sample after the initial application of the flame. t_2_—the total burning time of the sample after the second application of the flame. t_3_—the glow time of the sample after it is extinguished following the second application of the flame.

## Data Availability

The original contributions presented in this study are included in the article. Further inquiries can be directed to the corresponding author.

## Abbreviations

The following abbreviations are used in this manuscript:

APP	Ammonium polyphosphate
HDT	Heat deflection temperature
IFR	Intumescent flame-retardant system
LOI	Limited oxygen index
MO	Metal oxide
PER	Pentaerythritol
pHRR	Peak heat release rate
THR	Total heat release
UL94V	Underwriters Laboratories flammability vertical test for plastics
